# Patient and community nurse perspectives on recruitment to a randomized controlled trial of urinary catheter washout solutions

**DOI:** 10.1002/nop2.285

**Published:** 2019-04-14

**Authors:** Ashley Shepherd, Emma Steel, Anne Taylor, William Gordon Mackay, Suzanne Hagen

**Affiliations:** ^1^ Faculty of Health Sciences and Sport University of Stirling Stirling UK; ^2^ Institute of Healthcare Policy and Practice, Health, Nursing and Midwifery University of the West of Scotland Paisley UK; ^3^ Nursing, Midwifery and Allied Health Professions Research Unit Glasgow Caledonian University Glasgow UK

**Keywords:** older people, randomized controlled trial, recruitment, retention, urinary catheter

## Abstract

**Aim:**

To give evidence around the acceptability of a proposed randomized controlled trial (RCT) of catheter washout solutions.

**Design:**

A sample of senior community nursing staff (*N* = 7) were interviewed, and four focus groups with a sample of community nurses were conducted. Eleven semi‐structured face‐to‐face interviews were undertaken with patients using a long‐term catheter.

**Methods:**

An in‐depth qualitative study using a phenomenological approach was employed. This approach was suitable to explore the lived experiences of patients and gain their viewpoints and experiences.

**Results:**

Nurse participants raised concerns about the removal of washout treatment or increased risk of infection in relation to which arm of the trial patients were randomized to. There was concern that patients could get used to the increased contact with nursing staff. Six patients who agreed to participate cited personal benefit, benefiting others and a sense of indifference. Four patients were unsure about taking part and one declined. All cited concerns about negative implications for themselves.

## 1 INTRODUCTION

The randomized controlled trial (RCT) is largely accepted as the most robust research method to determine the effectiveness and cost‐effectiveness of healthcare interventions. However, recruiting participants to RCTs, especially those that might not be expected to derive any personal benefit, is often difficult. This is an even bigger problem amongst older people who often have poor physical health and impaired cognitive functioning, all factors which have a negative impact on not only people's willingness to participate in research but also their likelihood of remaining in a study till completion (Chatfield, Brayne, & Matthews, 2005).

## 2 BACKGROUND

McDonald et al. (2006) highlighted that between 1994–2002, only 31% of 114 UK multicentre trials funded by the UK Medical Research Council and UK Health Technology Assessment Programme achieved their original recruitment target. As such, there is now a greater interest in patient's perspectives on trial recruitment and participation prior to trial set up to improve recruitment and retention processes.

“Gatekeeping” by healthcare professionals (HCPs) may be an important issue to consider pre‐trial where researchers depend on clinical colleagues to identify potentially eligible participants. White, Gilshenan, and Hardy (2008) describes gatekeeping as the reluctance on the part of HCPs to contribute patients for research studies and suggests that their lack of support may undermine a trial recruitment prospects. Others have noted that patients may be more willing to enter trials than expected by the HCPs caring for them as they may find some reassurance in their participation helping the advancement of knowledge and improving care for others (Ross & Cornbleet, 2003; Slevin et al., 1995).

We report a study that aimed to explore the trial‐related factors that might determine either a patient's decision to participate or a community nurse's decision to refer a patient to a trial to examine the effectiveness of urinary catheter washout solutions.

### 2.1 Proposed RCT

The proposed trial discussed with participants and community nurses aimed to address the effectiveness of urinary catheter washout solutions. Long‐term urinary catheters (LTCs) are used in over 90,000 people in the UK. Over half of LTC users have an underlying neurological condition, and the proportion of users with non‐neurological conditions rises with age (Gage, Avery, Flannery, Williams, & Fader, 2017). LTCs are a leading cause of infection and are associated with significant mortality and morbidity. With an increasing number of individuals living with LTCs, largely due to an ageing population, appropriate and effective management is essential.

Recurrent problems with LTCs are extremely common. Prevalence rates of 70% for catheter‐associated urinary tract infection (UTI) have been reported and 33% for catheter expulsion or dislodgement (Wilde et al., 2010). These problems can lead to significant pain and distress for the patient, unplanned callouts, unnecessary admission to accident and emergency units and ultimately 2,100 deaths in the UK each year (Feneley, Hopley, & Wells, 2015; Mackay et al., 2017). Such problems are most commonly caused by a blockage in the catheter resulting in urine leakage around the side of the catheter (bypassing), or alternatively urine is retained in the bladder causing painful bladder distention (Stickler, 2014).

Catheter washout solutions are commonly used in the UK to treat blockages, with figures from Scotland between 2001–2014 showing a 188% increase in the number of catheter washout solutions prescribed (ISD Scotland, 2017). The rationale for the use of catheter washout solutions is, however, unclear. A recent Cochrane review concluded that there was insufficient good quality evidence about the use of catheter washout solutions to guide clinicians as to their benefit or indeed associated harm. This was largely due to issues of poor recruitment and retention in the included trials (Shepherd, Mackay, & Hagen, 2017).

The main question this study aimed to address was how acceptable patients and community nurses were to the proposed RCT. Secondly, we wanted to identify what issues were likely to arise in recruiting to and retaining participants in a RCT with a catheter washout versus no catheter washout design.

## 3 METHOD

This qualitative study was conducted using a phenomenological approach. This approach was suitable to explore the lived experiences of patients and gain their viewpoints and experiences using semi‐structured interviews and focus groups. Interviews and focus groups were digitally recorded, transcribed verbatim and analysed using Braun and Clarke's six phases of thematic analysis.

### 3.1 Sample

This study was conducted in two phases. Phase 1: A purposive sample of senior community nursing staff (*N* = 7) was recruited from seven NHS health boards across Scotland. The seven health boards were selected to ensure a diverse geographical spread, ensuring a sample of both urban and remote areas, where community care provision may differ. The senior community nurse in each health board was contacted, and these nurses were fully informed of the study and given contact details for the research team should they have any questions. Nursing staff completed a consent form before interview participation.

Phase 2: Responses from phase 1 informed the selection of three health boards for phase 2. These areas were purposively chosen to include a range of catheter washout treatment regimens and urban and rural geographical areas. The senior nurses who participated in phase 1 of the study were asked to select community nursing teams in their health board area that the research team could approach. The nursing staff in these teams were asked to participate in one focus group (Table 1).

**Table 1:** Health board characteristics

Board ID	Location (Urban/Rural)	Catheter washouts routinely administered (Yes/No^a^)	Number of focus groups (no. of participants per group)	Patient interviews (*N*)
A^b^	Rural	Yes	1 (*N* = 5)	4
B^b^	Urban	No	1 (*N* = 4)	3
C^b^	Urban/Rural	No	2 (*N* = 9, *N* = 4)	4
D	Rural	Yes		
E	Rural	No		
F	Rural	No		
G	Urban/rural	No		

a Although washouts not routinely used, a small number of patients receive regular washouts, either by patient request or clinical recommendation.

b Selected for nurse focus groups and patient interviews.

The nursing teams were asked to select patients in their care who met our inclusion criteria (community patients aged 16 years or older who were long‐term users (more than 28 days) of an indwelling urethral or suprapubic catheter who had experienced at least one episode of blockage or bypassing). Information about the study was given to patients via their community nurse. Patients were asked to contact the researcher if they wanted to participate or needed any more information. The eleven patients who contacted the researcher all participated in the study.

Consolidated criteria for reporting qualitative research (COREQ): a 32‐item checklist for interviews and focus groups was followed throughout this study.

### 3.2 Data collection

Initial telephone interviews with senior nursing staff were undertaken by an experienced male researcher (WGM) who is employed as a lecturer in a School of Nursing. All other interviews and focus group discussions were led by one of the two research fellows (RFs) employed on the study (ES & AT). Both RFs were female, very experienced in qualitative research and qualified to Masters or PhD level. Neither of the RFs had met any of the patient participants prior to the study commencing. One RF had met some of the community nurses from one of the health boards prior to the study but only in her capacity as a nurse lecturer. Both RFs had an interest in continence care and care of the older person.

The proposed RCT design discussed with participants in this study was to randomize patients with a history of catheter blockage into either a treatment arm where washout solutions would be given once every week, or a control arm where no washout would be given.

- Treatment group
  o Weekly catheter washouts (with whichever solution, saline or citric acid, used in that specific health board area).
  o Re‐catheterization at 12 weeks (as per manufacturers’ guidelines) or sooner if required, for example, if a catheter washout has been given and the catheter was still blocked.
- Control group
  o No catheter washouts.
  o Re‐catheterization at 12 weeks or sooner if required, for example, if the catheter is blocked.

Phase 1: Semi‐structured telephone interviews with one senior community nurse from each of the seven health boards were conducted by WGM. The nurses were asked to discuss the acceptability or limitations of the proposed trial design. Further questions were asked pertaining to local current practice with regard to the management of LTCs in the community, the use of catheter washout solutions, any protocols guiding practitioners’ routine practice and actions taken when urinary catheter blockage occurs in a community setting.

Phase 2: 11 semi‐structured face‐to‐face interviews with patients using a LTC with at least one episode of blockage and four focus group discussions with community nurses were conducted to elicit participants’ potential willingness to participate in such a trial. A semi‐structured interview guide was used to ensure that the relevant questions related to trial participation and acceptability were posed. The patient interviews, which took place in the patient's home, started with an open question such as: “Tell me a little bit about when you first had your catheter fitted. Why did you need a catheter?” Focus group sessions were held in the community centres of the different health boards. The researcher started by asking community nurses: “What is your role in catheter care. What issues or problems do you encounter with patients using long‐term catheters?” For both patient interviews and focus groups, the interviewer then discussed the proposed RCT design in this study. The different treatment regimens employed when urinary catheter blockages occur in a community setting, including patient and staff experiences and preferences, were also discussed. The range of treatment options currently in use for urinary catheter blockages was discussed with patients so they were equipped with the information necessary to knowledgeably discuss the acceptability and feasibility of participating in the hypothetical trial.

The following recruitment scenarios were discussed during the interviews and focus groups:

- Scenario 1: Potential participant receiving regular catheter washouts at the time of recruitment and is allocated to control group.
- Scenario 2: Potential participant receiving regular catheter washouts at the time of recruitment and is allocated to treatment group.
- Scenario 3: Potential participant not receiving regular catheter washouts at the time of recruitment and is allocated to control group.
- Scenario 4: Potential participant not receiving regular catheter washouts at the time of recruitment and is allocated to treatment group.

### 3.3 Analysis

All interviews and focus group discussions were digitally recorded and transcribed verbatim by an independent external transcriber. Transcripts were analysed by ES based on Braun and Clarke's six phases of thematic analysis (Braun & Clarke, 2006). The data were discussed in depth by ES, AS, WGM and AT at phases one (data familiarization) and four (reviewing themes) of data analysis, prior to the production of a final report. QSR NVivo qualitative data analysis software was used to manage the organization and analysis of the data (QSR International Pty Ltd, 2015).

### 3.4 Ethics

All participants were given verbal and written information about the study and told that participation was voluntary. Confidentiality and the right to withdraw at any time were assured. Research Ethics Committee approval for this study was granted by the East of Scotland Research ethics group, REC ref 16/ES/0120.

## 4 RESULTS

In phase one, seven senior community nursing staff from separate health boards across Scotland were interviewed. In phase two, 11 patients were interviewed face to face. In some instances, a family member who was also an informal carer, also participated in the patient interview. Four focus groups (four, four, nine and five nursing staff participants in each group) with a total of 22 community nurse participants (Table 2) were held across three health board areas. Each focus group contained staff from one health board area.

**Table 2:** Characteristics of patients who participated in interviews (*N* = 11)

Patient ID	Board ID	Gender	Age	Conditions(s) which led to insertion of catheter	Catheter type	Regular catheter washouts received	Trial participation decision
1	A	Male	88	Enlarged prostate	Urethral	Yes	Unsure
2	A	Male	75	Stroke; *transient ischaemic attacks*	Urethral	Yes	Agree
3	A	Male	75	Stroke	Urethral	Yes	Agree
4	A	Male	82	Amputation (uniped)	Urethral	No	Agree
5	B	Female	68	Multiple sclerosis	Suprapubic	Yes	Unsure
6	B	Female	56	Paraplegia	Urethral	No	Agree
7	B	Male	66	Secondary colorectal cancer	Urethral	No	Agree
8	C	Female	57	Multiple sclerosis	Suprapubic	Yes	Unsure
9	C	Female	73	Paraplegia	Suprapubic	No	Disagree
10	C	Male	63	Multiple sclerosis	Suprapubic	Yes	Unsure
11	C	Female	55	Stroke; pneumonia	Urethral	No	Agree

### 4.1 Nursing perspective on conducting a RCT of urinary catheter washout solutions

Nurses concerns fell into three main themes: the randomization of patients into groups, the removal of treatment deemed beneficial and the temporary increase in nurse contact time.

#### 4.1.1 Randomization of patients

From the perspective of the senior nurse participants and those nurses in the focus groups, the main issue about the trial design was in relation to the randomization of patients. While all potential participants in the proposed trial would have a history of catheter blockage, there would be variability in their existing catheter management plans. The nurses voiced concern that although patients may have received catheter washouts prior to trial commencement either on a regular basis, when their catheter blocked, or not at all, this could influence their willingness to take part.

#### 4.1.2 The removal of treatment deemed beneficial

Nurses were most concerned about Scenario 1, with a strong belief that you cannot cease the provision of regular catheter washouts to patients who are benefiting from them:

Board B Senior Community Nurse: I would presume then we might have difficulty recruiting if they think well there's a chance that I might be given nothing from the something I have just now. And then I suppose the question would be, OK, that's fine, I'll still participate. But then what are you going to do if I start having to get more frequent catheters and just how long will I be on the study? That's a valid thing I would be asking if I had to try something else and then I got problems with my catheter because of it.

About Scenario 4, the main concern amongst the nurses was that these participants could be at increased risk of infection or bladder irritation due to the washouts, thereby potentially creating a problem which would not have occurred outside the trial:

Board F Senior Community Nurse: I wouldn’t want to interrupt that closed system any more than is necessary, because obviously every time you do that there’s increased risk of infection.

Board A Focus Group: No I wouldn't want to do a weekly washout on a patient. That's just irritating their bladders. That's too much

#### 4.1.3 Temporary increase in nurse contact time

There was also concern that patients could get used to the increased contact with nursing staff that they would receive as a trial participant, which they could then miss if regular catheter washouts were not given following the trial period. The nurses highlighted that the participant information and consent would have to be clear and concise so that patients understood the length of the trial, with reminders throughout the duration of the trial. However, it was also discussed that there would be an argument for regular catheter washouts being maintained beyond the trial period for any patients who were benefiting from them:

Board B Focus Group: If that was working for that particular patient then there would be no rationale to take it away. You’ve got the evidence there to show that it’s been beneficial for them.

Scenario 3 did not raise any concerns with participants. For the boards where regular catheter washouts were currently not offered, recruiting into the control group would not be an issue as it would match their standard care. Likewise, there was no concern from a nursing perspective for patients recruited through Scenario 2. The only issue might be a change in the frequency of the catheter washout, but administering a weekly washout was not seen as an issue in relation to patient comfort or time and resources as many of these patients were currently visited on a weekly basis.

### 4.2 Patient perspective on participating in a RCT of urinary catheter washout solutions

#### 4.2.1 Reasons for agreeing to participate

Six patients hypothetically agreed to taking part in a future RCT (Table 2), regardless of which group they were allocated to control (no washouts) or treatment (weekly washouts). Reasons for agreeing to participate fell into three themes: perceived personal benefit, benefiting others and a sense of indifference.

##### Perceived personal benefit

Several patients identified ways they believed they would personally benefit from taking part in the study. These were related to their understanding of their current catheter care and options available to them when blockage occurs:

Interviewer: Would you have any issues getting a weekly washout as part of research?

Patient 6: No. You would know that you’re not going to get any sediment.

At the time of interview, Patient 6 was not receiving regular catheter washouts, but she had had a positive experience with them in the past for clearing sediment. She therefore viewed recruitment into the treatment group as being potentially beneficial to herself, reducing the number of blockage episodes. Despite the control group being no different to her current care, the opportunity to have the perceived benefit of regular catheter washouts encouraged her to agree to participate in the hypothetical trial.

##### Benefiting others

In interviews, patients explained that they would be motivated to take part in the study at least in part because they felt it may help others:

Interviewer: What if he was randomised into the group where we then said we’re going to have to stop the washouts and just change the catheter when there’s an issue. Would that concern you?

Family member of Patient 3: Not really, no. I mean if it’ll help somebody in the future, I think a lot of studies should have been done more on catheters to be honest… He’s had a really bad stroke and everything, but his catheter is his main problem, it causes him more distress than anything else.

As illustrated by Patient 3's family member, agreeing to participate in the trial was fuelled by a sense of altruism. Although living with multiple co‐morbidities, it was his catheter which caused Patient 3 the most distress as he suffered from frequent UTIs and blockages, despite receiving regular catheter washouts. As a result, leaving the house was almost impossible for Patient 3 due to the discomfort and concern of bypassing in public. Likewise, Patient 11 cited benefiting others as a motivation to participate in the trial, and a perceived personal benefit:

Patient 11: If it’s going to help an elderly person from pain and discomfort, yeah I would do it to help.

Interviewer: Does it worry you that you would maybe be put into the group that was getting a washout every week?

Patient 11: No it wouldn’t bother me, cause it would help my condition just now, with the sediment… If it’s going to help me I would agree to it, as long as I wouldn’t get an infection.

While Patient 11's initial inclination was to agree to the trial to help others, she also perceived the potential for personal benefit if recruited into the treatment group. Having never received a catheter washout before, her preconception was that regular washouts would be beneficial to her current situation rather than potentially disadvantageous.

##### Indifference

Interviewer: Would he be OK having weekly or fortnightly washouts do you think?

Family member of Patient 2: Yes, I'm quite sure he would be.

Interviewer: And what about if he was put into the other group where we change the catheter every time there was an issue, as opposed to using a washout solution, would that be an issue?

Family member of Patient 2: No I don’t think so. Well it’s a problem getting it in right enough. I mean there are odd times when they come and it’s not a problem at all and other times it’s awful.

Two patients hypothetically agreed to participation mainly because they could not perceive any active harms. While Patient 2 was receiving regular catheter washouts at the time of the interview, he was still experiencing frequent blockages. His wife therefore perceived no potential harm if he was to be recruited into the control group, other than the difficulty of inserting the catheter which he experienced on regular occasions anyway.

#### 4.2.2 Reasons for refusing to participate

Four patients were unsure about whether or not they would agree to participate in such a trial and one disagreed (Table 2). The main reason for an unwillingness to participate was the same across all five of these patients: concerns about negative implications for themselves.

#### 4.2.3 Negative implications of participation

All five patients who were either unsure or certain that they would not participate in the trial cited preference for a certain treatment. Of the four patients who were receiving regular catheter washouts at the time of interview, all were concerned about being randomized into the control group and therefore the withdrawal of washouts from their care:

Patient 5: I have found a difference since they’ve been coming in to flush it… As long as I knew that I would get my flush… I definitely think the flushes are, well, to me they’re definitely positive.

Patient 5 had been benefiting from regular catheter washouts and was concerned that being placed into the control group could potentially cause ongoing issues beyond the trial period. Likewise, Patient 8 had found relative success with regular catheter washouts and would be unlikely to participate in the trial if recruited into the control group:

Patient 8: It is scary to think (about the control group). To be perfectly honest I couldn’t live without the washouts. Because I can see that it actually does break down, you can actually see it all coming out.

Patient 9 was the only one to disagree to participate in a future trial. Unlike the four patients who were unsure about their decision to participate, Patient 9 was not receiving regular catheter washouts at the time of interview. Her personal concern was that being recruited into the treatment group and receiving a weekly catheter washout would be “a little bit, kinda much” on top of managing her other complex health issues.

## 5 DISCUSSION

This study has highlighted the potential challenges of recruitment and retention to a RCT of catheter washout solutions from the perspectives of both patients and nurses. Community nurses expressed the view that there was a need for such a trial and potential benefits; however, they were concerned about changing a patient's current catheter care if that was seen to be working. Nurses also raised concern about possible harm that they believed might result from those recruited to the treatment group including increased infection and bladder irritation. Loss of decision‐making powers, clinical autonomy and the inability for health professionals to personalize patient care has been reported in several trials as a reason for poor recruitment (Taylor, 1992; Taylor, Margolese, & Soskolne, 1994) and was a concern raised by nurses in this study too. As was the fear of feeling responsible if patients did not receive the intervention that turned out to be the most effective. This has been reported to affect health professionals' decisions to take part in trials (Taylor et al., 1994). The possible negative consequences of randomization to either group and a change to normal care were voiced by half of the patients interviewed.

Clinical equipoise has been proposed as the solution to concerns about randomization in clinical trials. This was defined by Freedman (1987) as an uncertainty in the medical community about the relative clinical merits of the intervention arms in a trial. Where insufficient evidence exists to judge one intervention in a trial as inferior to others, it means that health professionals can randomize patients without violating their duty of care because there is genuine uncertainty about what is best (Arras et al., 2014). A patient can then enrol in a trial without having to worry about being knowingly disadvantaged. In this study, clinical equipoise was missing as many of the nursing staff and patients had clear views about the benefits of the catheter treatment arms proposed.

Perceived personal benefit was as a motivating factor for participation for several participants. Patients for whom a current healthcare treatment is not working well are likely to regard trial participation as a positive opportunity to access a new intervention that may benefit them more (Snowdon, Elbourne, & Garcia, 1998). It would be important in this trial to emphasize that participants may not receive any benefit at all. Without this realization, there could be a large dropout rate if participants subsequently feel they are not benefiting from taking part in the trial.

Several patients interviewed in this study expressed a willingness to participate with both the perceived personal benefits from trial participation and the altruistic motivation influencing the decision to participate. This is not dissimilar to many other studies that have described people's reasons for trial participation as a “win‐win” situation—where they could help others and benefit personally (McCann, Campbell, & Entwistle, 2010).

Overall, the main challenge to recruitment of patients in the proposed catheter washout trial was that many saw no personal benefit to participation, with some even indicating a potential harm such as an increased risk of infection with the regular use of washout solutions. Some patients were reluctant to agree to participate if their current catheter management care was working for them. Nurses also said that if a patient was happy with their catheter care regimen and this had the potential to be changed as a result of the trial, people would be less likely to participate.

Patient preferences for a particular treatment and worry about uncertainty of treatment are commonly cited barriers to participation in RCTs. The employment of parallel non‐randomized patient preference groups enables patients who refuse randomization to participate in the group of their choice (Preference Collaborative Review Group, 2008). This recruitment strategy allows patients who are benefiting from regular catheter washouts at the time of recruitment to choose the treatment group if they wish, thereby having as little impact as possible on their catheter management plan in the constraints of the RCT. Or, in the instance of Patient 9 who believed that weekly catheter washouts would be too intrusive on top of managing her other health issues, she would be given the choice to participate in the control group. Under this study design, patients without a strong preference for the control or treatment group would still be randomized, leading to a four‐armed trial (Brewin & Bradley, 1989). This type of design, as described by McCann et al. (2010), has been used to aid recruitment in several healthcare trials. Often the number of participants recruited to the preference arms is restricted and once that quota is reached, patients can only participate in the randomized arms of the trial.

In recruiting to a trial such as the one proposed in this study, we have to take into account the complex needs of the patient group in question, that is, generally an older population, with co‐morbid conditions. A large number of RCTs exclude or have a low retention of older patients and patients with multiple co‐morbidities, leading to the external validity and overall generalizability of their results being questioned (Fortin et al., 2006). Previous RCTs and Cochrane reviews of catheter‐related trials have concluded that they were generally unsatisfactory, largely due to poor recruitment and retention. Moore et al. (2009) described considerable difficulties with recruitment of patients to her catheter washout trial, with study numbers falling far short of target. Also, maintaining participants in catheter‐related trials has been difficult for several reasons including death, ill health, catheters removed during the trial and request by nursing staff for their patients to be removed from trials (Kennedy, Brocklehurst, Robinson, & Faragher, 1992; Moore et al., 2009; Muncie, Hoopes, Damron, Tenney, & Warren, 1989). Therefore, obtaining an adequate sample for the catheter washout RCT proposed is likely to require a large multicentre trial.

Nurses identified that increased contact with participants would be seen by many as a potential motivation to participate in this trial. Marcantonio et al. (2008) concluded in their study of older adults that the chance to socialize with staff or other study participants was one of the main motivational factors to study participation. Others have suggested that older patients are more likely to accept risk in a study to have more interaction with healthcare staff, more attentive medical care and greater social interaction. The nurses in our study said it would be important to ensure that the information sheet and consent form were very clear in terms of the length of the trial and that those receiving weekly visits, would only do so for the duration of the trial. Also, a clear discussion before consenting a patient to such a trial would help clarify an individual's reason for research participation.

### 5.1 Strengths and limitations

The strengths of this study include the detailed interviews with a cross section of patients who would be eligible to participate in the actual trial. Using open‐ended questions allowed us to gain an insight into patients’ views and experiences. Also, by including the perspectives of the nurses caring for these patients, we were able to explore their views on participation which would be crucial if nurses were delivering the care requested of the trial and were also potential gatekeepers to trial participants. One important caveat of our study was that nurses and patients were recruited from Scotland only, and any definitive trial would most likely require recruitment from a large number of sites across the UK; however, a diverse sample of health boards was selected. To improve trustworthiness of the data, methodological triangulation was used by gathering data by means of different data collection methods. Investigator triangulation was applied by involving several researchers as research team members and involving them in addressing the organizational aspects of the study and the process of analysis. Member check was, however, not undertaken largely due to the demands on participant's time to undertake this.

## 6 CONCLUSION

Given the challenges of achieving adequate recruitment in trials, pre‐trial studies such as this are essential to develop optimized recruitment and retention procedures. From this study, specific recruitment and retention issues have been identified. Patients and nurses were largely supportive of the proposed trial; however, the need to offer a patient preference group in any future RCT may be necessary. Recognizing the facilitators and barriers to research participation is important to nurses who often act as the “gatekeepers”. As clinicians or researchers, nurses need to take into account the needs of older patients with co‐morbid conditions when participating in research.

## CONFLICT OF INTEREST

No conflict of interest noted for any author.
